# Palatal Wound Healing with Primary Intention in a Rat Model—Histology and Immunohistomorphometry

**DOI:** 10.3390/medicina56040200

**Published:** 2020-04-24

**Authors:** Liat Chaushu, Marina Rahmanov Gavrielov, Gavriel Chaushu, Marilena Vered

**Affiliations:** 1 Department of Periodontology and Implant Dentistry, School of Dental Medicine, Tel-Aviv University, Tel-Aviv 69978, Israel; dr.marindent@gmail.com; 2 Department of Oral & Maxillofacial Surgery, School of Dental Medicine, Tel Aviv University, Tel-Aviv 69978, Israel; gabi.chaushu@gmail.com; 3 Department of Oral & Maxillofacial Surgery, Rabin Medical Center, Campus Beilinson, Petah Tiqwa 49100, Israel; 4 Department of Oral Pathology, Oral Medicine and Maxillofacial Imaging, School of Dental Medicine, Tel Aviv University, Tel-Aviv 69978, Israel; lmy@netvision.net.il

**Keywords:** wound healing, primary intention, stem cells

## Abstract

Background and objectives: Subepithelial connective tissue graft (SCTG) from the palate has been considered as the “gold standard” for the treatment of deep gingival recessions. A single-incision technique was reported to allow primary wound healing. A palatal single incision was performed in a rat model. The present study assessed the histology and histomorphometry of palatal wound healing following surgical closure with primary intention. Materials and Methods: Twenty-six 6-month-old male Wistar rats weighing 427–650 g. An incision was made on the maxillary palate. A full thickness flap was raised palatally, and then repositioned and sutured. Two experimental groups: S—Study group, I—Intact control group. Half of the animals were sacrificed 7 days and the remaining 14 days postoperatively. Outcome parameters included—epithelial gap; inflammatory infiltration; vascular fraction, expression of myofibroblasts and stem cell markers within the oral epithelium and stromal cells and physical properties of stromal collagen fibers. Investigations were performed at two time-points (7 and 14 days) during the wound healing process. Results: The epithelial gap closed completely after 14 days. The inflammatory reaction and vascular fraction were relatively low. Surgical trauma downregulated the expression of cytokeratin (CK) 14 and CK 15, which returned to normal after 14 days. Epithelial differentiation was mediated through upregulation of connective tissue sex- determining-region-Y-box2 (SOX2). Epithelial SOX2, CD34, alpha smooth muscle actin (αSMA) and physical properties of stromal collagen fibers were not influenced by the surgical trauma. Conclusions: Surgical trauma followed by palatal wound healing with primary intention in a rat model heals within 14 days. It induces minimal inflammatory infiltration and vascular proliferation. Epithelization is exerted through promotion of epithelial differentiation from stem cells by connective tissue SOX2.

## 1. Introduction

Recent studies advocate the subepithelial connective tissue graft (SCTG) as the “gold standard” for soft tissue augmentation, especially, deep gingival recessions [1,2,3,4,5]. Harvesting SCTG from the palate is accompanied by substantial inconvenience. Various clinicians described a diversity of surgical techniques intended to lower this morbidity.

Hürzeler and Weng [6] presented a single-incision technique, followed by primary intention closure. Primary wound healing was thought to minimize donor site discomfort. Different harvest surgical techniques were compared. Primary wound closure following a single-incision technique was regarded as less invasive and less painful [7,8,9,10].

The remaining part of the connective tissue at the donor site following harvest was mentioned as another factor that may encourage post-operative wound healing [11,12].

The mucosa protects the oral cavity by being a barrier against intruders. When its integrity is compromised, either by acute or chronic challenges as surgical procedures, the body initiates a multistep complex healing process at the injured site [13,14,15,16,17]. Cells, both epithelial and mesenchymal, play an important role in the healing cascade [18,19,20,21,22,23].

Recently, the field of regenerative medicine introduced several blood-derived components ((i) platelet-rich plasma (PRP), (ii) platelet-poor plasma (PPP), (iii) platelet gel (PG), (iv) platelet-rich fibrin (PRF), (v) serum eye drops (E-S) and (vi) PRP eye drops (E-PRP) [24]) to promote wound healing. Their common action is PG enriched with local growth factors (vascular-endothelial growth factor (VEGF), platelet-derived growth factor (PDGF), transforming growth factor beta 1 (TGF-β1) and other) potentiating stem cells [24]. This became popular for the treatment of skin ulcers, radiodermatitis and other conditions [25,26,27]. Recently, a case of life-threatening oral mucositis (OM) following high-dose conditioning chemotherapy for peripheral blood stem cell transplantation (PBSCT), which was successfully treated with cord blood platelet gel (CBPG) was described [28]. Furthermore, a statistically significant improvement of OM following PG treatment was shown in patients treated with chemo- and radio-therapy [24].

To the best of our knowledge, there is lack of information in the literature assessing microscopical events following palatal wound healing surgical closure by primary intention. The aim of the present study was to assess the histology and histomorphometry of primary soft tissue healing, following palatal surgical incision, in a rat model, via expression of mesenchymal and epithelial cell markers.

## 2. Materials and Methods

### 2.1. Animals and Preparation of Experimental Model

Twenty-six 6-month-old male Wistar rats weighting 427–650 g were involved in this study. All experiments were conducted in accordance with the Guidelines on Animal Experiments in Tel Aviv University after approval by the Tel Aviv University Animal Care Committee.

**Figure 1:**
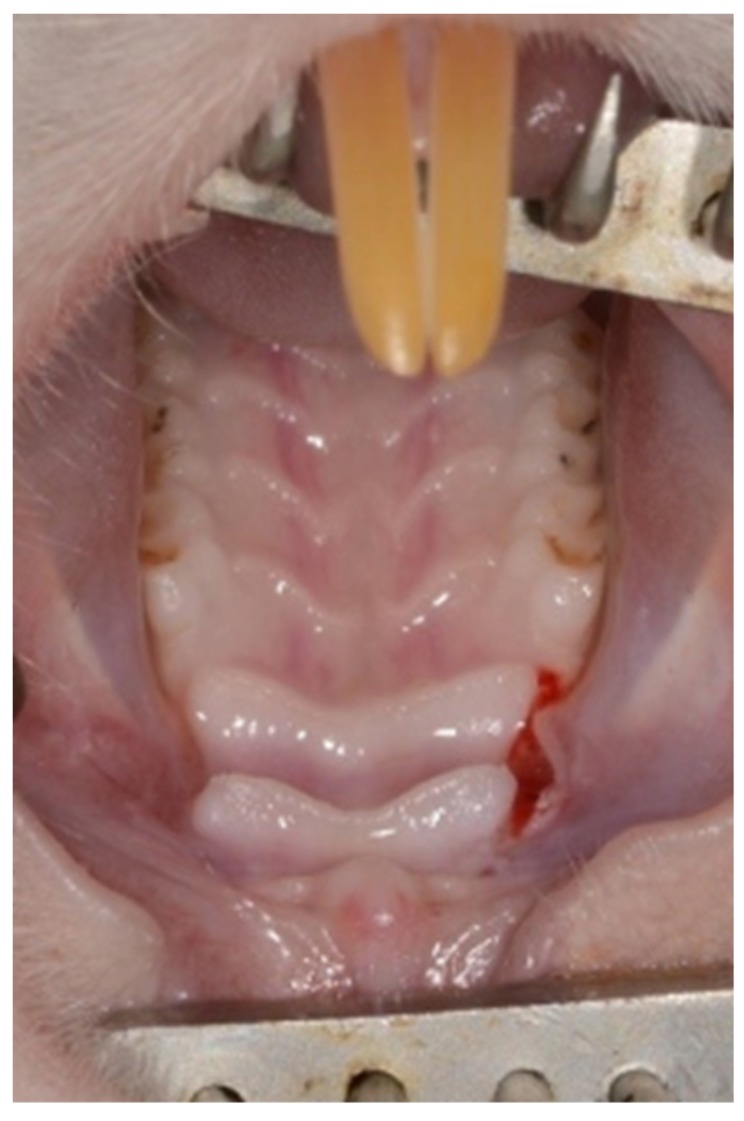
Palatal incision.

General anesthesia was achieved by an intraperitoneal injection of 0.1% Xylazine (10 mg/kg) and 0.1% Ketamine (80 mg/kg). In addition, Isoflurane (Terrell^TM^) was administered by a vaporizer at 2%–3% 100–200 mL/min combined with oxygen supply. Local anesthesia was performed using 0.4 mL Lidocaine 0.5% with adrenaline 1:100,000. A palatal incision was made on the maxillary alveolar ridge in the existing edentulous area between the 1st molar and the incisors using a 15c blade (Figure 1).

**Figure 2:**
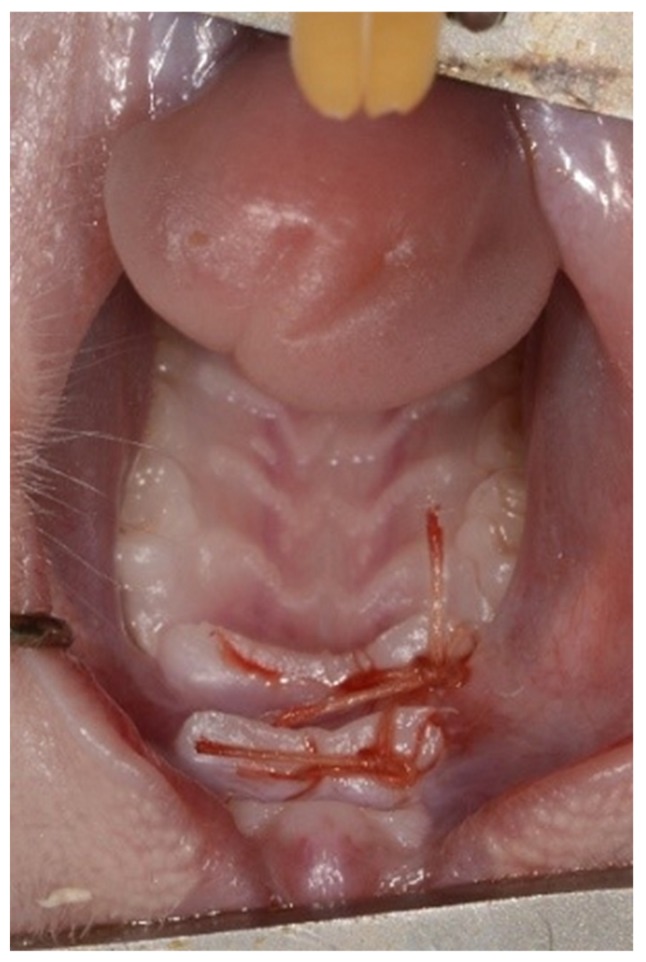
Primary closure with resorbable sutures.

A full thickness flap was raised bilaterally and was then repositioned and sutured with resorbable Vicryl Rapid^TM^ 5-0 sutures (Ethicon Inc., Johnson & Johnson, NJ, USA) (Figure 2).

### 2.2. Experimental Groups

Animals were randomly attributed to 2 experimental groups with two postoperative time frames of 7 and 14 days:

**S**—Study group—Single incision, periosteal exposure, closure by primary intention.

**I**—Intact control group—No surgery performed.

Half of the animals were sacrificed 7 days and the remaining 14 days postoperatively. Day 7 post-incision corresponds to the end of the inflammatory-to-peak proliferation phases of the wound healing process and day 14 corresponds to the end of the proliferation and beginning of remodeling phase [13,14,15,16,17,29].

### 2.3. Tissue Harvesting

Animals were sacrificed following standard ethical guidelines using CO_2_ inhalation. The maxillae were excised and fixed in 4% paraformaldehyde for 7 days at room temperature. After decalcification in a 10% ethylenediaminetetraacetic acid (EDTA) solution for 30 days, (EDTA solution was replaced every 3 days), the specimens were embedded in paraffin and cut into serial sections of 4 µm thickness in a coronal plane, perpendicular to the line of incision.

### 2.4. Histological and Immunohistochemical Evaluation

Segments of the treated area were processed for histological and immunohistochemical evaluation using the following staining methods:

#### 2.4.1. Hematoxylin & Eosin (H&E) Staining

Following an overnight incubation at 60 °C, slides were washed in xylene (twice × 5 min) then transferred to decreasing ethanol concentrations (100%, 96%, 70%), washed in double distilled water (DDW) (twice) and stained by Harris Hematoxylin (Merck KGaA, Darmstadt, Germany). After washing in tap water, slides were shortly dipped in 1% acid alcohol and consequently washed in water. Finally, a 1-min staining in 1% Eosin (Merck KGaA, Darmstadt, Germany) was performed, followed by washes in DDW, ethanol (70%, 96%, 100%) and cover slipping.

##### Epithelial Gap Measurement

**Figure 3:**
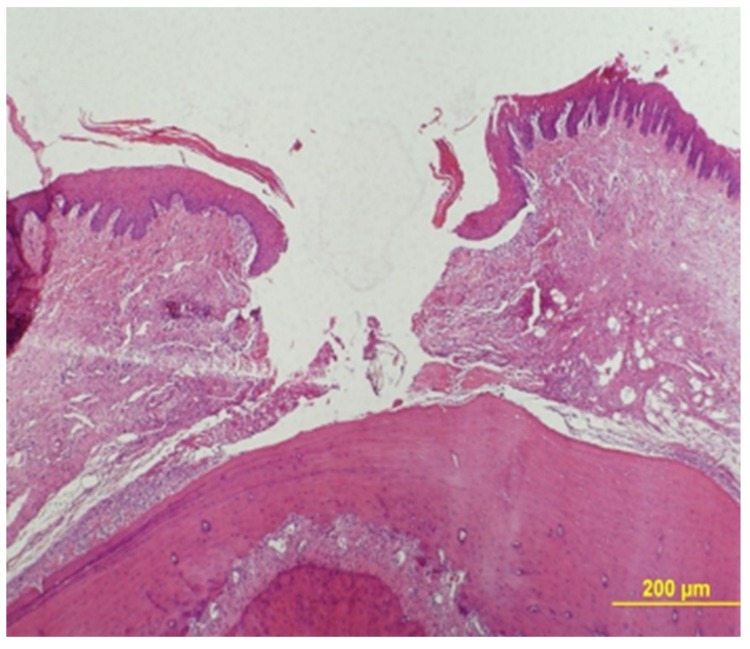
H&E staining for measurement of epithelial gap distance (×40).

Epithelial gap was measured under a ×40 magnification using an Olympus BH-2 light microscope (Olympus, Tokyo, Japan). The gap zone was defined by lack of wound tissue and its width was measured between two parallel lines that were drawn tangentially to the tissue that bordered the gap at its both sides (Figure 3).

A mean of all measurements in 2 serial sections were calculated. The results are presented as the mean ± standard deviation (SD) for each of the two time frames (7 and 14 days) of the study groups.

##### Histomorphometry (Inflammatory Infiltration, Vascular Density)

**Figure 4:**
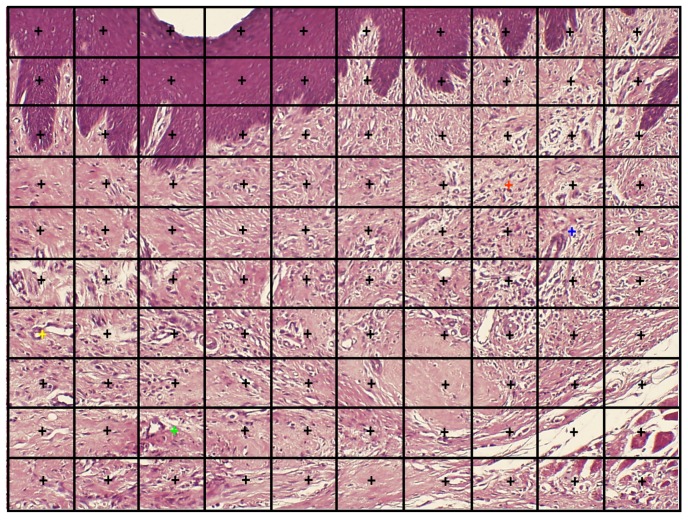
An example of the point-counting method. The "+" overlying the oral lining epithelium at the top of the photomicrograph should be excluded from counting. The yellow "+" overlies a blood vessel, therefore this parameter will be attributed 1 point. The green "+" is only adjacent to a blood vessel, therefore this parameter will score 0 points for the blood vessels. The orange "+" overlies an inflammatory cell (gain of 1 point), the blue "+" is only adjacent to inflammatory cells (0 points) (H&E, original magnification ×100).

Histomorphometry (light microscope, Olympus BH-2, Tokyo, Japan) was performed on a transversal plane of the maxilla. The histomorphometric method was an adaptation of the point-counting procedure [30]. In practice, selected fields were photomicrographed (JPEG format) at ×100 using a camera (Olympus DP70, Tokyo, Japan) with the wound area being positioned in the center of the field and the surface epithelium being positioned at and in parallel to the upper photographed field. Then, the JPEG photomicrograph was transported to a full PPT slide dimension. A 10 × 10 square-grid overlapped on top of each PPT slide. Point counting was performed on inflammatory infiltration and vascular density. Whenever the graticule-square center (marked by a "+") hit the parameter, it scored one point (Figure 4). The sum of points of the parameter in each case (Pi) was calculated and expressed as the area fraction percentage (AFP) as part of the total number of "+" summed in all sections that represent the whole section area (Σi) [31,32]. The results were represented as mean AFP for inflammatory infiltration and vascular density in each group.

#### 2.4.2. Immunnohistochemical Staining

Following an overnight incubation at 60 °C, slides were washed in xylene (twice × 5 min) and then, in declining concentrations of ethanol (100%, 96%, 70%), rinsed twice in DDW and placed for 10 min in 3% H_2_O_2_ for inactivation of endogenous peroxidases. Antigen retrieval was performed for each antibody as recommended by the manufacturer (Table 1). Pap pen-marked slides were washed in DDW and wash buffer (Zytomed systems DAB530, Berlin, Germany) in order to set pH at 7.0–7.4. Next, sections were treated for 40 min with background buster (Innovex biosciences NB306-50, Richmond, USA) in a humid chamber for minimizing nonspecific staining. Next, slides were incubated with a series of primary antibodies (Table 1).

Secondary antibodies followed for 30 min. Colorimetric reactions were performed using diluted diamino benzydine (DAB) chromagen (Zytomed systems DAB530, Berlin, Germany). Finally, slides were shortly dipped in Mayer’s Hematoxylin (Scy Tek labratories, Logan, U.S.A), washed in increasing ethanol concentrations (96%, 100%) and absolute xylene, and coverslipped. Positive tissue controls are detailed in Table 1; negative controls were achieved by omitting the primary antibody.

#### 2.4.3. Immunohistomorphometry

##### CK14 and CK15

The epithelial width was divided into the basal and upper halves at ×20 magnification. The immunoreaction was semi-quantitatively assessed and scored as staining intensity: 0—no staining, 1—weak staining, 2—intermediate staining, 3—strong staining. The mean staining intensity for the two halves per animal was calculated and the results were presented as the mean staining score per study group.

##### SOX2 (Nuclear Staining)

From each animal, two fields comprising of oral lining epithelium at the incision site were microphotographed at ×200 and similarly, the corresponding connective tissue fields beneath each of the epithelial fields. All microphotographs were saved as JPEG files and then copied to a full power point template (PPT) slide. A 100-square grid overlapped on the PPT slide. The number of positively-stained nuclei were counted in each field. Each case was given a mean score for the epithelial and stromal compartments, and each group was represented by a general mean of staining score (epithelium and stroma separately).

##### CD34

Immunohistomorphometry was performed by the aid of AFP, with a modification in the method used for the inflammatory infiltrate. Specifically, the assessment was performed at ×100 magnification with a 25-square grid that was placed over the incision area beneath the epithelial lining. If the "+" of the squares overlapped positively stained blood vessel walls or their lumina or any CD-34-positively stained cell/s within the connective tissue, a score of 1 was allocated to the case. The results were represented as mean AFP for CD34+ blood vessels/cells in each study group.

##### αSMA

Immunohistomorphometry was performed at ×100 magnification with a 25-square grid, in a modified manner compared to that of CD34. Since αSMA is expected to be positive in the smooth muscle cells in blood vessel walls as well as in wound healing-related myofibroblasts, each of the 25 squares was given an individual score for the overall expression of this marker: 0—no expression, 1—a few scattered positive cells/blood vessels, 2—abundant cells/blood vessels but not throughout the entire square, and 3—abundant cells/blood vessels all over the examined square. The mean of scores for all 25 squares was calculated per each animal. The results were presented as the general mean score for each study group.

##### Histochemical Analysis of Collagen Fibers

The physical properties of the collagen fibers were analyzed using picrosirius red (PSR) stain and polarized microscopy [33]. Tissue sections, 8-μm thick, were prepared and stained for 30 min with Sirius red (0.1% of Sirius red in saturated aqueous picric acid), for collagen bundle staining. Sections were studied by a light microscope under polarized light (Olympus BH-2, Tokyo, Japan), on which a digital camera was mounted (Olympus DP-70, Tokyo, Japan). Photomicrographs were saved as JPEG files that were subsequently copied onto power-point (PPT) slides, each slide showing side-by-side the original and the 90°-rotated counterpart picture. A grid composed of 10 doted parallel lines, with an equal distance between any 2 following lines, was superimposed on each picture. Those collagen bundles that overlapped the grid lines on the original picture were identified and their color was recorded and then compared to the counterpart, 90°-rotated picture. Changes in polarization colors of the collagen bundles between the original to the rotated picture were recorded: green-to-yellow, green-to-orange/red, and yellow-to-orange/red and vice versa. In addition, events of disappearance or appearance of polarization colors that were generated by the 90° rotation were also recorded. The percent of those collagen bundles with changed colors from the total number of bundles was calculated for each case. The results were presented as the mean percent number of collagen fibers with change of color for each study group.

### 2.5. Statistical Analysis

Data were entered and analyzed in SPSS version 24. First, descriptive statistics were produced, while means and standard deviations were calculated for all continuous measures. Outcome indices between groups (S, I), along time (7 and 14 days) were tested using two-way analyses of variance (ANOVA). Significant differences between groups were further examined using post-hoc analyses (Bonferroni tests). A *p*-value lower than 5% was considered to be a significant result.

## 3. Results

Twenty-six animals survived the end of the study and were included in the statistical analysis (Table 2), S (*n* = 16), I (*n* = 10).

### 3.1. Epithelial Gap

Epithelial gap (Table 3) decreased significantly between 7 and 14 days (*p* < 0.01) in the S group.

### 3.2. Inflammatory Infiltrate

A significant difference was found between experimental groups (*p* = 0.004). The inflammatory infiltrate was significantly increased in the S vs. the I group on days 7 and 14 (Table 4). Inflammatory infiltrate decreased from day 7 to 14 in the S group, however, differences were not significant (*p* > 0.05).

### 3.3. Vascular Density

No significant difference between experimental groups (Table 4). No significant interaction was found between groups and time.

### 3.4. CK14 Expression

**Figure 5:**
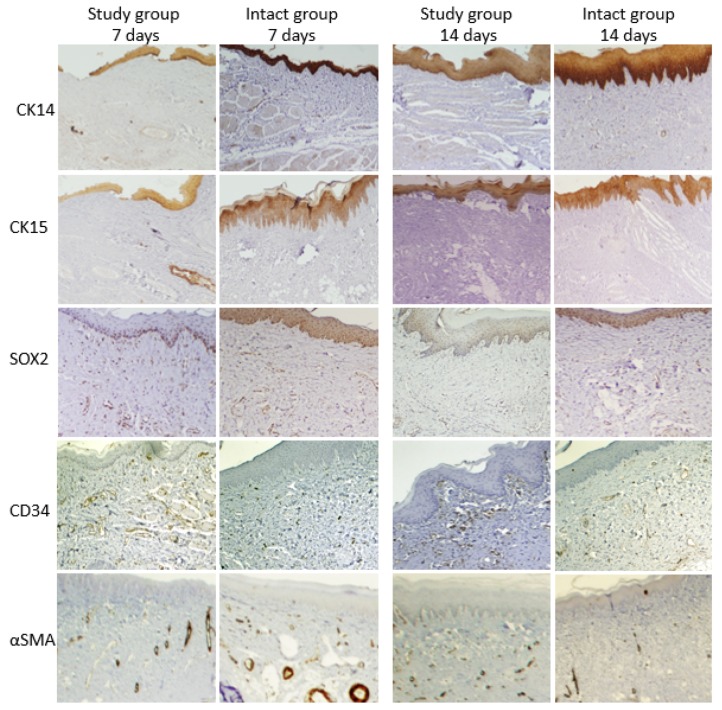
A panel of representative fields for the various examined markers in the Study and Intact groups, at 7- and 14-days postoperative time points.

A significant difference between experimental groups (*p* < 0.001) was found (Table 5, Figure 5). Expression of CK14 was significantly lower only after 7 days in the S group (*p* < 0.01).

### 3.5. CK15 Expression

A significant difference between groups (*p* < 0.001) was found (Table 5, Figure 5). Expression of CK15 was significantly lower after 7 days in the S group and remained low even after 14 days.

### 3.6. Epithelium and Connective Tissue SOX2 Expression

#### 3.6.1. Epithelial SOX2

No significant difference was found between groups (Table 6, Figure 5). Means in the S group were close to those in the I group. No significant interaction was found between group and time.

#### 3.6.2. Connective Tissue SOX2

A significant difference was found between groups (*p* < 0.001) with a higher expression in the S group (Table 6, Figure 5). Similar levels were found in each group at 7 and 14 days.

#### 3.6.3. CD34 Expression

**Figure 6:**
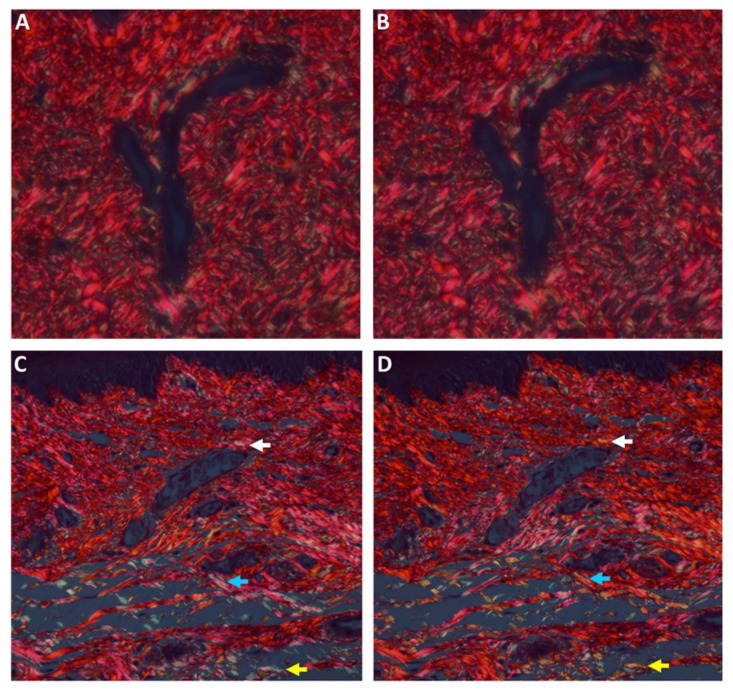
Histopathological sections of picrosirius red-stained collagen fibers seen with polarized light. A and B represent the same field seen at the original position (**A**) and then rotated 90° (**B**). The polarization colors do not change after rotation. C and D represent the same field seen at the original position (**C**) and then rotated 90° (**D**). Arrows point to collagen fibers that changed polarization colors after rotation. (A–D, original magnification ×400).

No significant difference between groups (Table 7, Figure 5), αSMA (Figure 5), PSR (Figure 6). No significant changes along time.

## 4. Discussion

The present study evaluated the effects of single incision trauma on primary wound healing. Histologically, it was demonstrated that the epithelial gap almost closed completely only after 14 days. The mechanism through which this effect is exerted was further evaluated. It can be speculated that due to primary wound closure, the inflammatory reaction was relatively low and no statistically significant changes occurred along time. It can be inferred that the inflammatory reaction was initiated by the incision, but due to primary closure it remained low. Similarly, no changes were noted in vascular density indicating a lack of granulation tissue.

CK14 is recognized as a specific epithelial marker, particularly of the basal cells [34,35,36]. The increased values in the S group at day 14 support the early appearance of K14 in the differentiation process. CK14, in addition to it being associated with basal cell proliferation, is also known for its role as a maintainer of basal cell shape, provider of resistance to mechanical stress and messenger of signals coming from the adjacent connective tissue cells [37].

CK15 is another specific marker of the basal epithelium [38,39]. The low values in the S group at day 14 support the late appearance of K15 in the differentiation process. Some studies showed that like CK14, CK15 can also form a filament network of the cytoskeleton, and as such, acts in a similar way to that of CK14 [40].

SOX2 is a member of the SOX family of transcription factors [41]. SOX2 may have important roles in the formation of early pluripotent embryonic cells [42]. Surgical trauma did not affect the expression of epithelial SOX2. Connective tissue SOX2 was upregulated following surgical trauma.

CD34 is expressed in hematopoietic progenitor cells of myeloid and lymphoid lineage and stromal cells in several organs [43]. αSMA is a marker of myofibroblasts. PSR demonstrates collagen maturation. None of these factors was significantly influenced by the surgical incision.

Primary wound closure following single-incision technique has been regarded as less invasive and less painful [7,8,9,10,11]. Based on the results of the present study, it can be speculated that the minimal inflammatory infiltrate noted histologically and the fast wound closure could be responsible for reduced pain and morbidity in patients.

The remaining part of the connective tissue at the donor site following harvest was mentioned as another factor that may influence post-operative wound healing [11,12]. The upregulation of connective tissue SOX2 may explain the importance of the connective tissue in wound healing. The thicker the connective tissue remaining, the better wound healing will be established.

Future studies should also focus to identify what kind of clinically available growth factors (e.g., platelet gel [24,25,26,27,28]) may be used to improve the healing processes.

## 5. Conclusions

Surgical trauma followed by palatal wound healing with primary intention in a rat model heals within 14 days. It induces minimal inflammatory infiltrate and vascular proliferation. Epithelization may be exerted through promotion of epithelial differentiation from stem cells by connective tissue SOX2.

## Figures and Tables

**Table 1:** Antibodies used for immunohistochemistry in the study* **.

Target	Marker	Primary Antibody Manufacturer Dilution Time of Exposure Clone Catalog #	Positive Control Tissue
Actin	αSMA ***	Dako A/S, Denmark 1:100 1 h Clone 1A4 Catalog #M0851	Blood vessels
Embryonic stem cell	SOX2	Novus Biologicals, Littleton, U.S.A 1:150 1 h Polycolonal Catalog #NB110-79875	Normal human brain
Hematopoietic stem cells	CD34	Abcam, Cambridge, U.S.A 1:1000 1 h Clone EP373Y Catalog #ab81289	Human kidney
Epithelial cells	Cytokeratin (CK) 14	Proteintech, U.S.A 1:200 1 h Polycolonal Catalog # 10143-1-AP	Human lung cancer (Squamous)
	Cytokeratin (CK) 15	Proteintech, U.S.A 1:200 1 h Polycolonal Catalog #10137-1-AP	Human oesophageal cancer

* Antigen retrieval procedure for all the antibodies—EDTA pH 9%. ** Secondary antibody for all the antibodies—Zytomed HRP one step Polymer, Zytomed systems, Berlin, Germany. *** αSMA – alpha smooth muscle actin

**Table 2:** Summary of cases in the study.

		S	I	Total
**Days**	7	8	5	13
	14	8	5	13
	Total	16	10	26

**Table 3:** Epithelial gap (mm).

Group	Day	Mean	SD
**S**	7	0.76	0.92
	14	0.04	0.11
	Total	0.38	0.71

**Table 4:** Inflammatory infiltrate & vascular density (%).

		Inflammatory Infiltrate		Vascular Density	
Group	Day	Mean	SD	Mean	SD
**S**	7	27	23	9	5
	14	7	12	12	4
	Total	18	21	10	5
**I**	7	2	4	6	5
	14	2	3	10	5
	Total	2	4	8	5

**Table 5:** CK14 and CK15 intensity score.

		CK14		CK15	
Group	Day	Mean	SD	Mean	SD
**S**	7	1.3	0.4	1.3	0.3
	14	2.0	0.7	1.7	0.7
	Total	1.7	0.6	1.5	0.5
**I**	7	2.1	0.5	1.8	0.7
	14	2.7	0.3	2.2	0.3
	Total	2.4	0.5	2.0	0.5

**Table 6:** Epithelium and Connective Tissue SOX2 mean positive nuclei score.

		Epithelium		Connective Tissue	
Group	Day	Mean	SD	Mean	SD
**S**	7	147	53	56	28
	14	147	58	48	25
	Total	147	54	52	26
**I**	7	160	44	20	9
	14	136	13	14	6
	Total	148	33	17	8

**Table 7:** CD34 (%).

		CD34	
Group	Day	Mean	SD
**S**	7	17.5	9
	14	20.5	10
	Total	19.0	10
**I**	7	33.6	14
	14	25.6	13
	Total	29.6	13
